# Age and menopause affect the expression of specific cytokines/chemokines in plasma and cervical lavage samples from female sex workers in Nairobi, Kenya

**DOI:** 10.1186/1742-4933-10-42

**Published:** 2013-10-22

**Authors:** Aida Sivro, Julie Lajoie, Joshua Kimani, Walter Jaoko, Francis A Plummer, Keith Fowke, T Blake Ball

**Affiliations:** 1 Department of Medical Microbiology, University of Manitoba, Winnipeg, Manitoba, Canada; 2 Department of Immunology, University of Manitoba, Winnipeg, Manitoba, Canada; 3 Department of Medical Microbiology, University of Nairobi, Nairobi, Kenya; 4 National Microbiology Laboratories, Public Health Agency of Canada, Winnipeg, Manitoba, Canada

**Keywords:** Aging, Immune system, Cytokines/chemokines, Menopause

## Abstract

**Background:**

Aging of the immune system, known as immunosenescence, is associated with profound changes in both innate and adaptive immune responses, resulting in increased susceptibility to infection and a decreased ability to respond to vaccination. The purpose of this study was to investigate the effect of age and menopause on the expression of 22 different cytokines/chemokines in both plasma and cervical lavage samples from female sex-worker cohort from Nairobi, Kenya (age range 20–65).

**Results:**

Cytokine/chemokine levels were measured using a Miliplex multiplex assay (Millipore). We found that age positively correlated with MCP-1 (p = 0.0002) and IP-10 (p = 0.03) systemic cytokine expression, and that women over 50 expressed the highest levels of these cytokines, but also had elevated expression of MIG (ANOVA p = 0.0096) and MIP-3β(ANOVA p = 0.0434). We also found that IL-8 (p = 0.047) and sCD40L (p = 0.01) systemic expression negatively correlated with age. Further, MIG (p = 0.0081) and MCP-1 (p = 0.0157) were present at higher levels in post-menopausal women suggesting a potential estrogen dependant systemic regulation of these cytokines. In cervical lavage samples, age did not directly correlate with the expression of any of the tested cytokines/chemokines, however sIL-2Rα (ANOVA p = 0.0170) and IL-15 (ANOVA p = 0.0251)were significantly higher in women over 50. Menopause was shown to have a more profound effect on cytokine expression in the cervical mucosa with MIG (p = 0.0256), MIP-3α (p = 0.0245), IL-1β (p = 0.0261), IL-6 (p = 0.0462), IL-8 (p = 0.007), IP-10 (p = 0.0357) and MCP-1 (p = 0.0427) all significantly under-expressed in post-menopausal women.

**Conclusions:**

This study demonstrates that aging and menopause-associated hormonal changes are associated with significant changes in systemic and mucosal cytokine/chemokine expression, which may have implications for the age-related decline in the ability to fight against infections.

## Background

One of the great successes of modern society is the global increase in life expectancy; in part due to greater control of infectious diseases, better living conditions, better nutrition and new medical technologies [1-3]. Furthermore, the gap between the life expectancy of populations in developed and developing countries is decreasing. This increase in aging population is seen as a success story for socioeconomic development and public health policies; however it also poses new challenges on the health care system. In order to provide appropriate health care to the elderly, a better understanding of how age impacts the immune system and susceptibility to disease is needed.

Globally, increase in the number of elderly individuals has resulted in increased incidence of cancer and autoimmune disorders [3-7]. Importantly, aging is also associated with defects in the immune response to multiple infections [5,8,9]. In fact, aging has been associated with decrease in lymphocyte proliferative responses, reduced delayed type hypersensitivity reactions and decreased antibody responses to vaccination and infection [9-11]. Generally, increased age is characterized by a decreased ability to generate effective immune response to foreign antigens, which impairs the ability to fight against infections. It is generally believed that this occurs due to a decrease in function and number of naïve T cells [11-15]. The ratio of naïve to memory T cells has been shown to decrease with age, due to the fact that most naïve T cells have been previously exposed to antigens and fewer T cells are produced in the thymus [3,11,13-16]. Defects in T cell signaling in both CD4+ and CD8+ subsets have been described [11,17-19]. Additionally aging has been associated with higher number of T regulatory (Treg) cells [5,18-21], a decline in B cell production and humoral immunity [21-24], chronic immune activation [23,25,26] and a shift from Th1 [Interleukin (IL-)2, Interferon-gamma (IFN-γ)] to a Th2-like cytokine response (IL-4, IL-6, IL10) [26,27]. All these data suggest that the ability of an older individual to respond appropriately to an immunological challenge is compromised thorough variety of mechanisms.

Age associated alterations in immune environment have predominantly been studied in systemic compartments and far less is known about the impact aging has on the mucosal immune responses. The elderly have been characterized with higher rates of morbidity and mortality due to infectious diseases of the intestinal tract and a general mucosal immunosenescence [18]. This is evidenced by findings showing that the development of mucosal immune response to new antigens at the gastrointestinal tract (GI) diminishes with age [19]. However, studies looking at aging in women and the associated changes in immune environment of the mucosal compartment, especially the cervical mucosa, are currently lacking.

In addition to aging, the onset of menopause, and the associated decline in estrogen levels play a critical role in regulating the immune response in women. Hormones are immune regulators that can modify both the systemic and mucosal immune system [28,29]. Important changes in cell populations and cytokine/chemokine production are observed during the menstrual cycle at the female genital tract (FGT) [29]. After menopause there is an increase in pro-inflammatory serum markers such as IL-1, IL-6 and TNF-α, a decrease in CD4 T and B lymphocytes and a decrease in the cytotoxic potential of natural killer (NK) cells [11].

Since cytokines and chemokines play an important role in modulating the immune system it is important to understand how their expression might change with age. In this study, we look at the impact of age and menopause on the systemic and cervical mucosal expression of 22 cytokines/chemokines. To our knowledge this is the first study to look at the impact of both age and menopause on cytokines and chemokines important in immune cell trafficking and inflammation in both systemic and mucosal compartments.

## Results

### Study participants characteristics

Only HIV-ve individuals were used in this study. Additionally, because other sexually transmitted diseases can greatly influence the expression of cytokines/chemokines at the systemic and mucosal level, all participants who tested positive for the presence of bacterial vaginosis, *Nisseria gonorrhoeae*, and/or *Chlamydia* were excluded from the analysis (n = 26). Overall 176 subjects were enrolled, resulting in 176 plasma samples and 154 CVL samples (where available) that were included in the final analysis. Due to sample volume limitations, this was the case for all tested cytokines/chemokines except MIP-3β and IL-12p70 (assayed for n = 79 for both plasma and CVL). Age range for patients where plasma samples were analyzed was 20 to 65 (median age: 38) and age range for patients providing CVL samples was 21–65 (median age: 39). Other socio-demographic characteristics were similar between the compared groups (not shown).

### Plasma cytokine/chemokine levels

**Figure 1:**
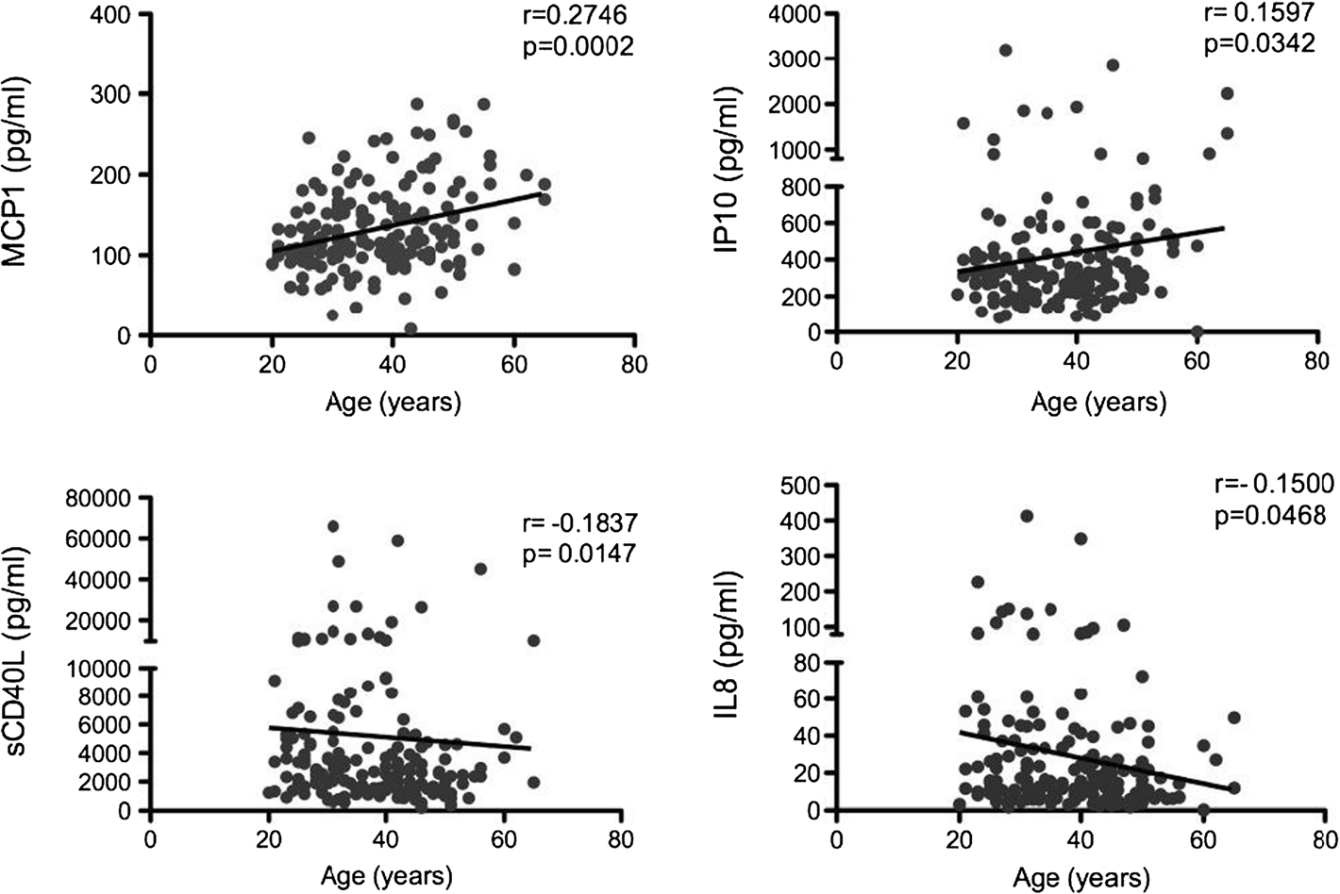
**Spearman correlation of age (years) and plasma cytokines/chemokines concentration (pg/ml).** Only statistically significant correlations were shown for the rest of the data refer to Additional file 1: Tables S1 and S2. Linear regression line was added for visualization purposes only.

**Figure 2:**
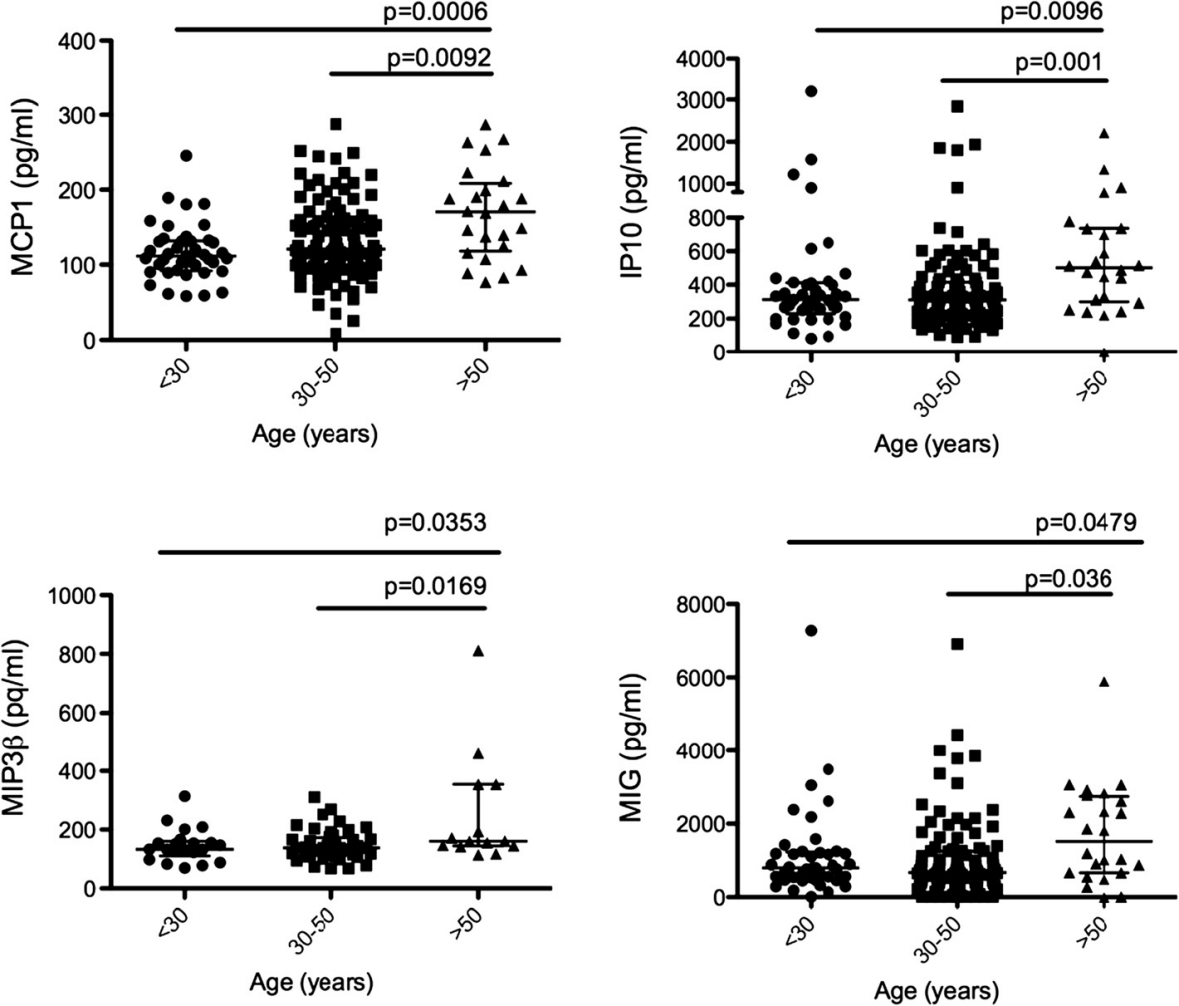
**Plasma cytokine/chemokine expression (pg/ml) grouped by age: younger (<30), middle-aged (30–50) and older (>50) women.** Overall significance was determined using a Kruskal-Wallis test, and intergroup differences were examined using Mann–Whitney test (p < 0.05).

To determine what cytokines and chemokines were altered due to age we correlated the expression of each specific cytokine/chemokine examined with the subject’s age using Spearman correlation analysis (Additional file 1: Table S1). We observed that the expression of MCP-1 (p = 0.0002) and IP-10 (p = 0.03) positively correlated with age. In contrast we found that the systemic expression of IL-8 (p = 0.047) and sCD40L (p = 0.01) negatively correlated with the age of the women studied (Figure 1). To better understand the distribution of differences in cytokine expression based on age, we subdivided the data into the following age groups: young (<30 years), middle-aged (30–50 years) and old (>50 years) as done elsewhere [30]. We found that MCP-1 and IP-10 levels were highest in women above 50 years of age compared to the other two age groups (ANOVA p = 0.026 and p = 0.004 respectively, Figure 2). Interestingly we found that MIP-3β (ANOVA p = 0.0434) and MIG (ANOVA p = 0.0096) were also highly expressed in women over 50 (Figure 2). No significant differences were observed between <30 and 30–50 age groups for any of the above-mentioned cytokines, suggesting that the age effect on the expression of the pro-inflammatory cytokines/chemokines MCP-1, IP-10, MIG and MIP-3β is more pronounced in older women (> 50 years of age). Overall, these data show that MCP-1, IP-10, MIG, MIP-3β, sCD40L and IL-8 may be important indicators of age-related changes in the systemic immune environment.

**Figure 3:**
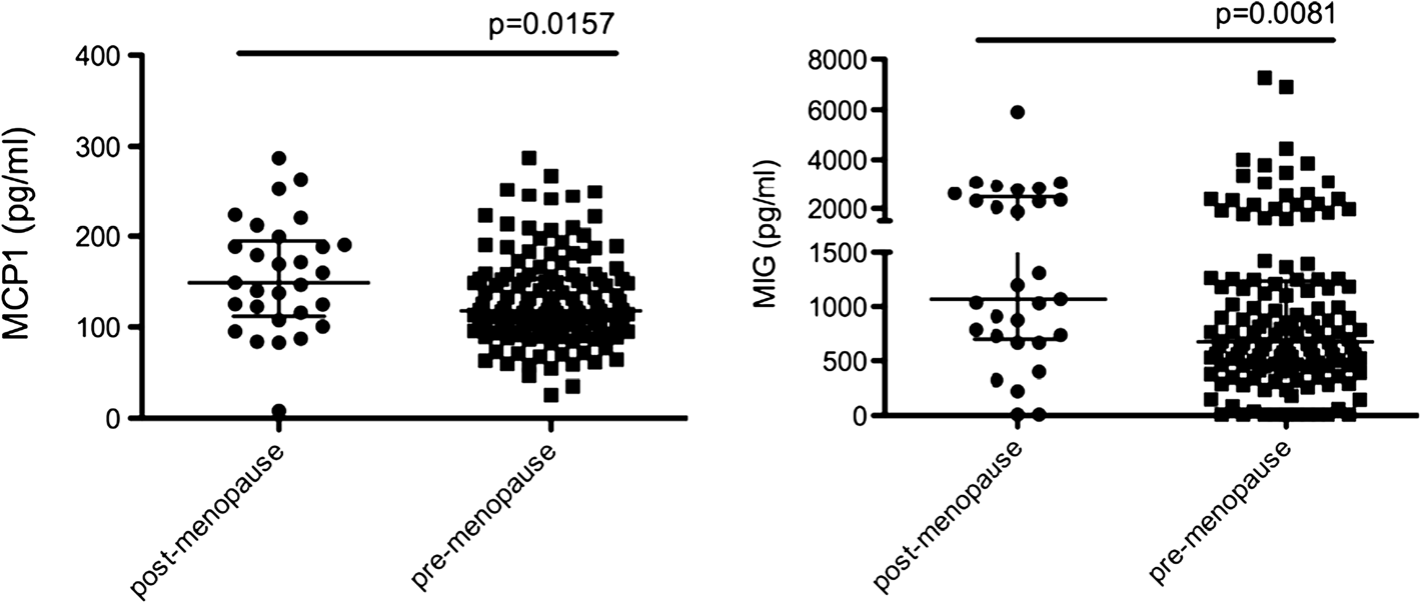
**Plasma cytokine/chemokine expression (pg/ml) in post-menopausal (n = 29) and pre-menopausal (n = 140) women.** Mann–Whitney test was used to examine significance (p < 0.05).

Studies suggest that in addition to age, changes in the immune environment can be attributed to the hormonal changes associated with menopause transition [11]. To determine the effect of menopause on the systemic expression of studied cytokines/chemokines we segregated the samples into pre-menopausal (n = 140) and post-menopausal (n = 29). Some of the studied women were menopausal before age of 50 (n = 9), and some women above 50 years of age were not menopausal (n = 4). When we examined the expression differences between these two groups we found that plasma levels of MIG (p = 0.0081) and MCP-1 (p = 0.0157) were significantly higher in post-menopausal women (Figure 3) suggesting a potential hormonal regulation of these cytokines. As these cytokines were also significantly affected by age, we ran linear regression models with MCP-1 and MIG as a dependent variable and age and menopause as covariates (Additional file 1: Table S3). Linear regression model indicates that MCP-1 is primarily affected by age (p = 0.004) but not menopause (p = 0.449), while MIG expression was significantly affected by menopause (0.049) but not age (p = 0.280).

### CVL cytokine/chemokine levels

**Figure 4:**
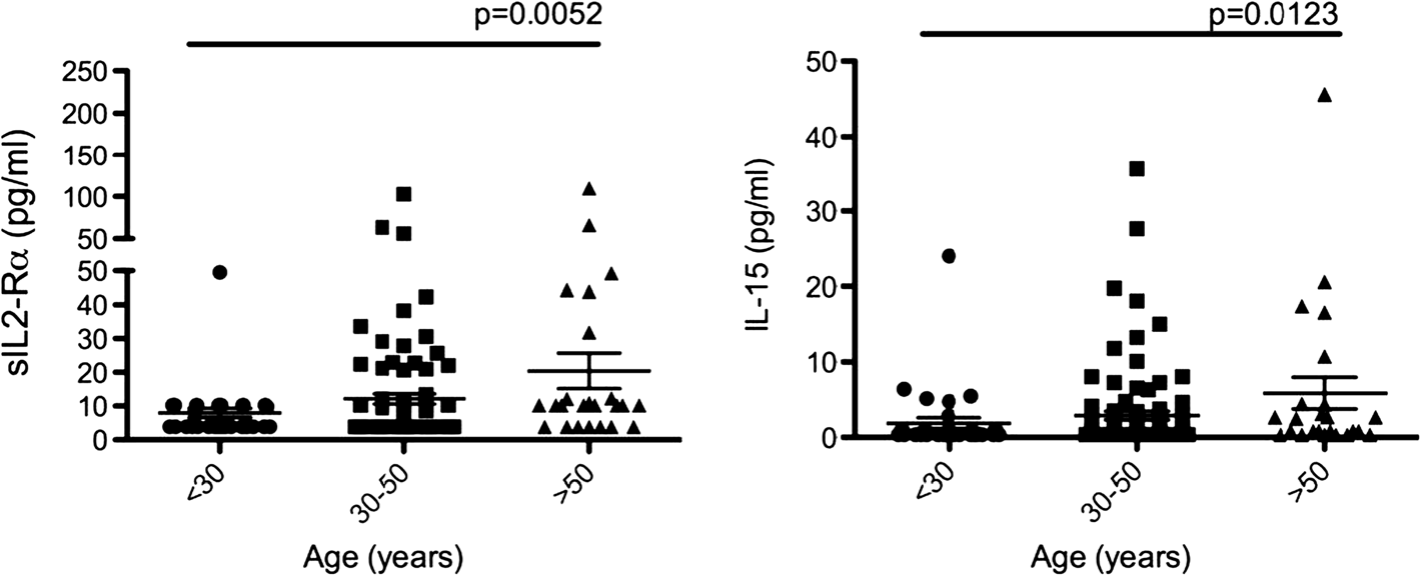
**CVL cytokine/chemokine expression (pg/ml) grouped by age: younger (<30), middle-aged (30–50) and older (>50) women.** Overall significance was determined using a Kruskal-Wallis test, and intergroup differences were examined using Mann–Whitney test (p < 0.05).

Despite recent advances in understanding the aging immune system, studies looking at the effect of aging on the immune environment of the mucosal compartment, especially the cervical mucosa, are lacking. To investigate the impact of aging on mucosal cytokine/chemokine expression, Spearman correlation analysis was used to correlate cervical lavage cytokine/chemokine expression with age. We observed no significant correlations between the mucosal expression of the studied cytokines/chemokines and women’s age (Additional file 1: Table S2). Again, we subdivided the women to examine if there were any differences in cytokine/chemokine expression between specific age groups (<30; 30–50; >50). We found that the expression of sIL-2Ra (ANOVA p = 0.0170) and IL-15 (ANOVA p = 0.0251) were significantly higher in women above 50 years of age compared to younger (<30) women (Figure 4). It would seem that the effect of age on the cervical immune environment in younger women is not as apparent and larger differences are observed in higher age groups, possibly due to physiological and behavioral factors.

**Figure 5:**
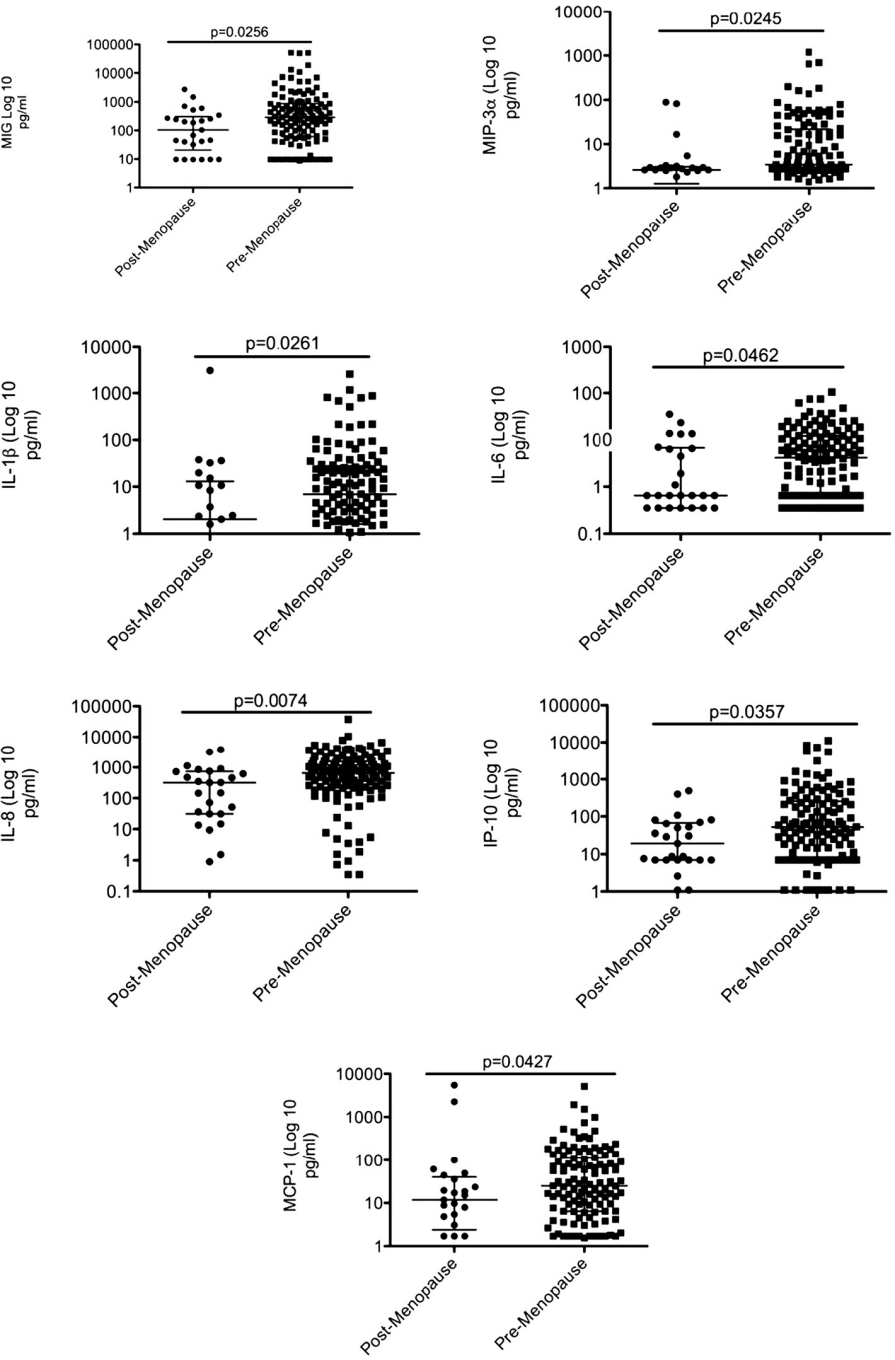
**CVL cytokine/chemokine expression (pg/ml) in post-menopausal (n = 29) and pre-menopausal (n = 135) women.** Mann–Whitney test was used to examine significance (p < 0.05).

Because estrogen deprivation is an important factor that can influence the cytokine/chemokine expression in the FGT, we next compared the cytokine/chemokine expression between pre-menopausal (n = 135) and post-menopausal (n = 29) women. Some of the studied women were menopausal before age of 50 (n = 5), and some women above 50 years of age were not menopausal (n = 4). We observed that the level of MIG (p = 0.0256), MIP-3α (p = 0.0245), IL-1β (p = 0.0261), IL-6 (p = 0.0462), IL-8 (p = 0.007), IP-10 (p = 0.0357) and MCP-1 (p = 0.0427) were all significantly elevated in women pre-menopause compared with women post-menopause (Figure 5). Our data shows a decrease in pro-inflammatory markers in cervical lavage samples of post-menopausal women and this could potentially contribute to higher susceptibility to microbial invasion and infection of the FGT observed post-menopause.

## Discussion

Aging is associated with deregulation and general decline in immune responses contributing to increased susceptibility to infection. Human–based data looking at the association between aging and immune response is particularly sparse, with majority of the studies published so far based on rodent models. Furthermore, limited data is available on the impact of aging and menopause on human cytokine/chemokine expression in different tissues. Herein, we showed an association between both age and menopause and cytokine/chemokine expression in plasma and cervical lavage samples from female sex workers in Nairobi, Kenya.

### Age related changes in plasma and CVL cytokine/chemokine expression

We observed a number of age related changes in plasma cytokine/chemokine expression. Systemic expression of MCP-1 and IP-10 positively correlated with women’s age. Consistent with our data, Seidler et al. showed that in healthy adults, serum concentration of MCP-1, but not MIP-1α, MIP-1β and fractalkine increased with age [30]. MCP-1, a chemoattractant for monocytes and lymphocytes mainly produced by endothelial cells and macrophages, is involved in the inflammation process [31] and is reported to be involved in Th2 polarization [32]. Increasing concentrations of MCP-1 in older individuals could partly be responsible for development of arthrosclerosis and an age dependant shift in Th1/Th2 responses [33]. IP-10 is an interferon-gamma inducible protein predominantly produced by monocytes and plays an important role in leukocyte trafficking by promoting the migration of NK cells and activated and memory T cells but not naïve T cells [33,34]. Interestingly, in elderly individuals the number of naïve T cells is low and it is conceivable that a higher concentration of IP-10 is required due to the higher number of memory T cells observed with age. Additionally IP-10 is a strong chemoattractant for Th1 lymphocytes and is considered to be a reliable marker of strong Th1- mediated autoimmune disease [35]. Our data agrees with the previous observations that an age-dependant increases in both MCP-1 and IP-10 chemokines in healthy women may affect both Th1 and Th2 phenotypes and contribute to the overall impairment of the immune system.

When we separated women into age groups (<30, 30–50 and >50) the expression of MCP-1 and IP-10 remained significant. Both MCP-1 and IP-10 were elevated in older women (>50 years old), suggesting that the expression of these 2 chemokines is predominantly associated with the higher end of the age distribution. Additionally, when we looked at the cytokine/chemokine expression in different age groups, MIG and MIP-3β were significantly higher in women aged >50. Interestingly, MIG and IP-10 belong to the same family of chemokines, suggesting that the IFN-gamma inducible pathway is influenced by age. To our knowledge this is the first study to show association of systemic MIP-3β and age. MIP-3β is produced by DCs and possibly other lymphocytes and has the ability to chemoattract T cells, B cells, macrophages and NK cells. MIP-3β could be one of the contributing factors in age dependant deregulation of the innate and adaptive immune responses.

Additionally we observed a negative association between age and IL-8 and sCD40L systemic expression. IL-8 is produced by macrophages and plays an important role in neutrophil recruitment. The percentage of T cells producing IL-8 has been shown to decrease with age in the acutely ill Malawian patients [36] and spontaneous production of IL-8 was found to be significantly lower in elderly male and females [37]. Our data agrees with the previous observations, suggesting that age is an important factor in IL-8 production. This age-dependant decrease in IL-8 production could contribute to the altered neutrophil function including reduced chemotaxis observed in older individuals [38]. Major sources of soluble CD40L are activated T lymphocytes and platelets. CD40L plays an important role in activation of antigen presenting cells (APCs) and regulation of B cell proliferation, adhesion and differentiation by interacting with its receptor, CD40. Significantly reduced CD40L expression by the aged CD4 T cells was observed in mice [39]. Lower CD40L expression was observed on lymphocytes from older subjects as well [40,41]. Aging has been previously associated with defects in platelet function [42] and this might contribute to decreased sCD40L production observed here. Consequently, this decrease in sCD40L could contribute to age related B cell deregulation [22].

To the best of our knowledge we are the first to examine the effect of age on the immune environment in cervical lavage samples. Interestingly, in this study no direct correlation was observed between the mucosal cytokine/chemokine expression and age overall. However, when we divided the women in three different age groups, we observed that the expression of sIL-2Rα and IL-15 was significantly higher in women above 50 years of age compared to younger women (<30). An increase in IL-15 mRNA was shown to be associated with aging in mouse model [9] and higher IL-15 serum levels were reported in ultralongeval individuals (>95) [43]. A relative increase in sIL-2Ra release by aged cells despite lower surface expression was reported previously [1]. IL-15 is known to play a role in T cell homeostasis and could contribute to the age associated decrease in naïve to memory T cell ratio [3-5] as well as increase in Treg cell numbers [5]. Additionally, increased IL-15 mRNA expression was observed in aging muscles in mice studies. This is thought to be an age-related adaptation of skeletal muscle to counter muscle atrophy through IL-15 mediated increase in myosin heavy chain protein content [9]. Aging and the associated muscle loss of the FGT could potentially contribute to increase in IL-15 observed here.

### Menopause related changes in plasma and CVL cytokine/chemokine expression

Menopause is associated with important physiological and immunological changes. The decline in production of estrogen associated with ageing in women has been linked with important changes in cell population and cytokine expression [11]. Keeping this idea in mind, we looked at the cytokine/chemokine expression in women depending on their menopause status.

In the systemic compartment, expression of MIG and MCP-1 were higher in post-menopausal women. MIG did not directly correlate with age however it was significantly higher in women above 50 years of age. Multivariate analysis indicates that MCP-1 is primarily affected by age, while MIG expression was primarily affected by the menopause status. It has been previously shown that MCP-1 expression is inhibited by estrogen therapy leading to decrease in macrophage recruitment [13-15]. Further studies are needed to determine weather increase in MIG and MCP-1 expression could in part be explained by menopause-associated decrease in estrogen levels.

Interestingly, when we looked at the impact of menopause status on the cytokine/chemokine expression in CVL we observed a higher impact when compared to plasma. Indeed, at the mucosal environment, it seems that the menopause associated estrogen deprivation has a bigger impact than age itself. Menopause status was significantly associated with lower mucosal expression of MIG, MIP-3α, IL-1b, IL-6, IL-8, IP-10 and MCP-1. Previous data showed an association of menopause with a decrease in T cell and B cell populations [11]. This decrease in the lymphocyte population may be cause, or effect of the altered mucosal cytokine/chemokine expression observed in our study. Our data seems to indicate that post-menopausal women might have a lower capacity to generate a strong, inflammatory mucosal immune response, which matches the findings from the GI mucosa [18,19]. Vaginal infections, especially yeast infections, are one of the main symptoms of menopause. Menopause is associated with physiological changes in the urogenital epithelium including thinning out, loss of elasticity and increase in vaginal pH [21]. These menopause-associated physiological changes in the FGT tissue result in increased ability of pathogens to establish infection. In addition to this, decreased pro-inflammatory cytokine/chemokine expression observed in our study could potentially contribute to increased susceptibility to microbial invasion and infection observed post-menopause. To our knowledge this is the first study to examine the effect of age and menopause status on cytokine/chemokine expression in CVL.

Some study limitations must be addressed. In this study we are looking at a specific population composed only of women age range 20–65 involved in sex worker cohort in Nairobi, Kenya, which might account for some of the difference between our study and others. Even if sex work has less or no impact on the systemic cytokine/chemokine expression, it is known to have an impact on the mucosal cytokine/chemokine expression dynamics (Lajoie et al., submitted). It is also important to note that p-values were discussed as observed without correction for multiple comparisons to avoid false negative results. Further investigations looking at changes in cell populations both systemically and mucosaly, are needed in order to fully understand the impact of age and menopause- associated hormonal changes on the immune environment.

## Summary

Our study demonstrates dynamic changes in systemic and cervical mucosa cytokine/chemokine expression during ageing in women. To the best of our knowledge this is the first extensive study that analyzed the impact of age and menopause status on cytokine/chemokine production in both the systemic and cervical mucosal compartment of African women. We have shown that significant differences exist in the way age and menopause-associated hormonal changes impact the systemic and mucosal cytokine/chemokine expression. Additionally, we have demonstrated that age and menopause impact on cytokine/chemokine expression differs between tissues highlighting the need for more specific tissue-based studies. Further understanding of how these changes affect susceptibility to infection and response to vaccination is crucial to the development of new therapies targeted at enhancing immune response in the elderly.

## Methods

### Study population

Samples used for this study were obtained from the Pumwani Sex Worker Cohort in Nairobi, Kenya established in 1985 [23]. Overall, 176 HIV-negative individuals were involved in this study. Participants of the cohort are assessed twice a year for follow-up. Prior to sample collection each woman enrolled in the study answered a questionnaire and the following information was collected: sociodemographic, sexual behavior, duration of sex work, number of sex clients, condom use, number of regular partners and reproductive history. Menopause data was obtained by self-report methodology. Participants also underwent HIV testing and a gynecological exam where vaginal specimens were obtained and tested for the presence of bacterial vaginosis (Nugent score), *Nisseria gonorrhoeae*, and *Chlamydia* (PCR). Informed written consent was obtained from all study participants. Ethics approval for this study was obtained from both the University of Manitoba and the University of Nairobi.

### Sample collection and processing

#### CVL

The cervix was washed with 2ml of sterile 1× phosphate-buffered saline (PBS) and the lavage was collected from the posterior fornix. Obtained samples were centrifuged and stored at −70°C. Samples were shipped to Winnipeg, Manitoba, Canada for analysis.

#### Plasma

Peripheral blood was collected in EDTA tubes. Plasma was collected and frozen at −70°C, and shipped to Winnipeg, Manitoba for further analysis.

### Cytokine/Chemokine measurement

Cytokine/chemokine levels were measured using Miliplex MAP multiplex kit (Human Cytokine/chemokine panel I and III from Millipore, Billerica, MA) and analyzed on the BioPlex-200 (Bio-Rad, Mississauga, ON, Canada). Human cytokine/chemokine panel I was analyzed following the overnight protocol whereas the human cytokine/chemokine panel III was analyzed following the 2h incubation protocol. Human cytokine/chemokine panel I included following cytokines: IL-1β, IL-2, sIL-2Rα, IL-6, IL-8, IL-10, IL-12p70, IL-15, IL-17, sCD40L, Fractalkine, IFN-γ, IP-10, MCP-1, MCP-3, MIP-1α, MIP-1β and TNF-α. Human cytokine/chemokine panel III included following chemokines: ITAC (CXCL11), MIG (CXCL9), MIP-3α (CCL20) and MIP-3β (CCL19). Samples with values below the lower detection limit (MinDC +2SD) were assigned the value half the lower detection limit in pg/ml, (MinDC + 2SD)/2.

### Statistical analysis

Statistical analysis was performed using the GraphPad Prism (version 5, GraphPad software, La Jolla, CA) and p values <0.05 were considered to be statistically significant. Its important to note that p-values are reported and discussed as observed and no corrections for multiple comparison were made [26]. Gaussian distribution was tested by D’agostino and Pearson’s omnibus normality test. Since our data did not follow normal distribution Spearman nonparametric correlation, Kruskal-Wallis and Mann Whitney non-parametric tests were used in the analysis. Multivariate analysis was performed using SPSS for Mac, version 20 (SPSS Inc., Chicago, Illinois, USA). Outcomes required log transformation to make the residuals behave properly.

## Abbreviations

CVL: Cervical lavage; HIV: Human immunodeficiency virus; FGT: Female genital tract; GI: Gastrointestinal tract; NK: Natural killer cell; DC: Dendritic cell; MCP-1: Monocyte chemoattractant protein 1; IP-10: Interferon gamma induced protein 10; MIG: Monokine induced by gamma interferon; MIP-3β: Microphage inflammatory protein 3 beta.

## Competing interests

The authors declare that they have no competing interests.

## Authors’ contributions

AS, JL, KF and TBB designed the project. AS and JL performed the experiments, analyzed the data and wrote the manuscript with critical review from all the authors. JK and WJ provided clinical and technical advice and assisted in subject recruitment and characterization. FAP, KF and TBB provided funds to support the project. All authors read and approved the final manuscript.

## Supplementary Material

Additional file 1 **Table S1.** Correlation of age and plasma cytokine/chemokine levels. **Table S2.** Correlation of age and CVL cytokine/chemokine levels. **Table S3.** Linear mixed models analyses to determine the effect of age and menopause on plasma MIG and MCP-1 expression in Kenyan FSW cohort. Click here for file
