# Supplementary material for: Age and menopause affect the expression of specific cytokines/chemokines in plasma and cervical lavage samples from female sex workers in Nairobi, Kenya
Source: Immun Ageing. 2013 Oct 22;10:42. doi: 10.1186/1742-4933-10-42 (PMC3874757; doi:10.1186/1742-4933-10-42)
Supplement: Additional file 1 — Table S1. Correlation of age and plasma cytokine/chemokine levels. Table S2. Correlation of age and CVL cytokine/chemokine levels. Table S3. Linear mixed models analyses to determine the effect of age and menopause on plasma MIG and MCP-1 expression in Kenyan FSW cohort. [file 1742-4933-10-42-S1.docx]

**ADDITIONAL FILE 1**

Table S1. Correlation of age and plasma cytokine/chemokine levels

| Cytokine/Chemokine | Spearman r | 95% confidence interval | P value (two-tailed) |
| --- | --- | --- | --- |
| MIP3β | 0.154 | -0.07619 to 0.3685 | 0.1755 |
| MIG (CXCL9) | 0.0794 | -0.07378 to 0.2289 | 0.2949 |
| MIP3α | -0.05838 | -0.2088 to 0.09472 | 0.4415 |
| ITAC (CXCL11) | 0.03457 | -0.1183 to 0.1859 | 0.6488 |
| IL1β | -0.079 | -0.2285 to 0.07416 | 0.2973 |
| IL2 | 0.1249 | -0.02785 to 0.2720 | 0.0985 |
| sIL2Rα | -0.08115 | -0.2306 to 0.0720 | 0.2843 |
| IL6 | -0.04703 | -0.1979 to 0.1060 | 0.5354 |
| IL8 | -0.1500 | -0.2956 to 0.002265 | **0.0468*** |
| IL10 | -0.01756 | -0.1694 to 0.1351 | 0.8171 |
| IL12p70 | 0.1293 | -0.1012 to 0.3466 | 0.2561 |
| IL15 | 0.0009025 | -0.1514 to 0.1531 | 0.9905 |
| IL17 | 0.04181 | -0.1112 to 0.1928 | 0.5817 |
| sCD40L | -0.1837 | -0.3268 to -0.03235 | **0.0147*** |
| Fractalkine (CX3CL1) | 0.08547 | -0.06768 to 0.2347 | 0.2594 |
| IFNγ | -0.01722 | -0.1690 to 0.1354 | 0.8205 |
| IP10 (CXCL10) | 0.1597 | 0.007659 to 0.3046 | **0.0342*** |
| MCP1 (CCL2) | 0.2746 | 0.1277 to 0.4098 | **0.0002***** |
| MCP3 (CCL7) | -0.1048 | -0.2530 to 0.04826 | 0.1664 |
| MIP1α (CCL3) | 0.0294 | -0.1234 to 0.1809 | 0.6985 |
| MIP1β (CCL4) | -0.01752 | -0.1693 to 0.1351 | 0.8175 |
| TNFα | 0.04736 | -0.1057 to 0.1982 | 0.5325 |

Table S2. Correlation of age and CVL cytokine/chemokine levels

| Cytokine/Chemokine | Spearman r | 95% confidence interval | P value (two-tailed) |
| --- | --- | --- | --- |
| MIP3β | 0.1576 | -0.1035 to 0.3983 | 0.2213 |
| MIG (CXCL9) | -0.09905 | -0.2598 to 0.067 | 0.2278 |
| MIP3α | -0.1170 | -0.2746 to 0.04664 | 0.1483 |
| ITAC (CXCL11) | 0.04621 | -0.1710 to 0.2591 | 0.6690 |
| IL1β | -0.1046 | -0.2635 to 0.0597 | 0.1980 |
| IL2 | 0.1340 | -0.03056 to 0.2914 | 0.0998 |
| sIL2Rα | 0.1243 | -0.04035 to 0.2824 | 0.1270 |
| IL6 | -0.07601 | -0.2359 to 0.08787 | 0.3488 |
| IL8 | -0.1043 | -0.2632 to 0.06004 | 0.1994 |
| IL10 | 0.1040 | -0.05983 to 0.2623 | 0.1994 |
| IL12p70 | 0.03958 | -0.2195 to 0.2935 | 0.7600 |
| IL15 | 0.1070 | -0.05675 to 0.2652 | 0.1864 |
| IL17 | 0.02108 | -0.1427 to 0.1838 | 0.7960 |
| sCD40L | 0.1175 | -0.06768 to 0.2948 | 0.1994 |
| Fractalkine (CX3CL1) | 0.07055 | -0.09441 to 0.2317 | 0.3878 |
| IFNγ | 0.06186 | -0.1031 to 0.2235 | 0.4490 |
| IP10 (CXCL10) | -0.05454 | -0.2154 to 0.1092 | 0.5017 |
| MCP1 (CCL2) | -0.004079 | -0.1673 to 0.1594 | 0.9601 |
| MCP3 (CCL7) | 0.06324 | -0.1006 to 0.2237 | 0.4359 |
| MIP1α (CCL3) | 0.04621 | -0.1180 to 0.2080 | 0.5706 |
| MIP1β (CCL4) | 0.03067 | -0.1328 to 0.1925 | 0.7057 |
| TNFα | 0.08488 | -0.07900 to 0.2443 | 0.2952 |

Table S3. Linear mixed models analyses to determine the effect of age and menopause on plasma MIG and MCP-1 expression in Kenyan FSW cohort.

|  |  | Beta | P-value | 95% CI | |
| --- | --- | --- | --- | --- | --- |
| lnMCP-1 | age | .290 | **.004** | .004 | .022 |
|  | menopause | -.075 | .449 | -.321 | .143 |
| lnMIG | age | -.109 | .280 | -.044 | .013 |
|  | menopause | .200 | **.049** | .003 | 1.503 |
